# The *Myxococcus xanthus* Two**-C**omponent System CorSR Regulates Expression of a Gene Cluster Involved in Maintaining Copper Tolerance during Growth and Development

**DOI:** 10.1371/journal.pone.0068240

**Published:** 2013-07-10

**Authors:** María Celestina Sánchez-Sutil, Juana Pérez, Nuria Gómez-Santos, Lawrence J. Shimkets, Aurelio Moraleda-Muñoz, José Muñoz-Dorado

**Affiliations:** 1 Departamento de Microbiología, Facultad de Ciencias, Universidad de Granada, Granada, Spain; 2 Department of Microbiology, University of Georgia, Athens, Georgia, United States of America; Centre National de la Recherche Scientifique, Aix-Marseille Université, France

## Abstract

*Myxococcus xanthus* is a soil-dwelling member of the δ–Proteobacteria that exhibits a complex developmental cycle upon starvation. Development comprises aggregation and differentiation into environmentally resistant myxospores in an environment that includes fluctuations in metal ion concentrations. While copper is essential for *M. xanthus* cells because several housekeeping enzymes use it as a cofactor, high copper concentrations are toxic. These opposing effects force cells to maintain a tight copper homeostasis. A plethora of paralogous genes involved in copper detoxification, all of which are differentially regulated, have been reported in *M. xanthus*. The use of in-frame deletion mutants and fusions with the reporter gene *lacZ* has allowed the identification of a two-component system, CorSR, that modulates the expression of an operon termed *curA* consisting of nine genes whose expression slowly increases after metal addition, reaching a plateau. Transcriptional regulation of this operon is complex because transcription can be initiated at different promoters and by different types of regulators. These genes confer copper tolerance during growth and development. Copper induces carotenoid production in a Δ*corSR* mutant at lower concentrations than with the wild-type strain due to lack of expression of a gene product resembling subunit III of *cbb3*-type cytochrome *c* oxidase. This data may explain why copper induces carotenoid biosynthesis at suboptimal rather than optimal growth conditions in wild-type strains.

## Introduction

Myxococcus xanthus is a model soil bacterium used to study a unique form of prokaryotic development. In the presence of nutrients, cells feed as coordinated groups. When starvation strikes, thousands of individuals cooperate to build a fruiting body inside of which the elongated cells differentiate into round, environmentally-resistant myxospores [1]. This complex developmental process must be accomplished in the presence of a fluctuating soil environment. In our laboratory, we dissect the global response to copper during the life cycle of M. xanthus. Copper, a metal widely distributed in nature, is essential for a variety of physiological processes in many forms of life. It is required by enzymes that utilize the Cu(II)/Cu(I) redox couple, such as cytochrome c oxidases, multicopper oxidases (MCOs), or Cu/Zn superoxide dismutases [2]. On the other hand, excess of copper may lead to metal substitutions, oxidation of proteins, and damage to membrane lipids and DNA. This dual effect forces the cells to maintain a precise copper homeostasis to guarantee protein function while avoiding metal toxicity. Any imbalance in these levels can be fatal [3], [4].

*M. xanthus* has evolved tight copper homeostatic control mechanisms, most of which are inducible by this metal. For this reason, while developing cells are more sensitive to copper than growing cells, cells from both stages reach the same level of metal resistance when cultures are pre-adapted to copper [5]–[8]. Carotenoids have been reported to accumulate in *M. xanthus* in the presence of copper to quench singlet oxygen generated by this metal. Blue light and copper are the only environmental agents known to induce carotenogenesis in *M. xanthus*. All the regulatory elements known to regulate carotenoid synthesis by blue light are also required for the response to copper, with the exception of CarF, which uniquely responds to light. Furthermore, induction of carotenoid biosynthesis by copper requires lower metal concentrations when cells are incubated at suboptimal growth conditions [5].

In addition to genes responsible for carotenoid synthesis, the M. xanthus genome contains many paralogous genes that are involved in the copper response [6]–[8]. Three families of paralogs related to copper and/or other metal homeostasis systems have been characterized: three members of the MCO family, three of the P_1B_-type ATPases family, and six members belonging to the CBA-efflux systems family [6]–[8]. Expression profiles of these genes have revealed that genes belonging to the same family are differentially regulated in response to copper and/or other metals. Accordingly, all these systems function sequentially to detoxify the cell compartment. Although genes involved in the copper regulon exhibit several types of expression profiles [6]–[8], for the sake of simplicity we refer here to only two of them. Some genes, such as those encoding the MCO CuoB and the P_1B_-type ATPase CopB, are part of an immediate response to copper as their expression rapidly increases after copper addition, reaching a peak by 2 h [6], [8]. Other genes, represented by those of the MCO CuoA and the P_1B_-type ATPase CopA, are involved in the maintenance of the metal response as their expression slowly increases after copper addition, achieving a plateau between 24 and 48 h of incubation with copper [6], [8].

Expression of this network of genes is orchestrated by diverse regulatory elements. We previously reported that CorE, the first member of a novel ECF (extracytoplasmic function) σ factor group, regulates genes involved in the immediate response [9]. The CorE regulon includes, among others, the P_1B_-type ATPase CopB and the MCO CuoB.

In this report, we identify an operon termed curA which comprises nine genes, including cuoA and copA. We demonstrate that the two-component system (TCS) CorSR, also encoded in this operon, regulates curA expression. Finally, we show that this cluster exhibits complex regulation and is involved in the maintenance of copper tolerance during growth and development.

## Results

### The Gene Cluster *curA*

**Figure 1:**
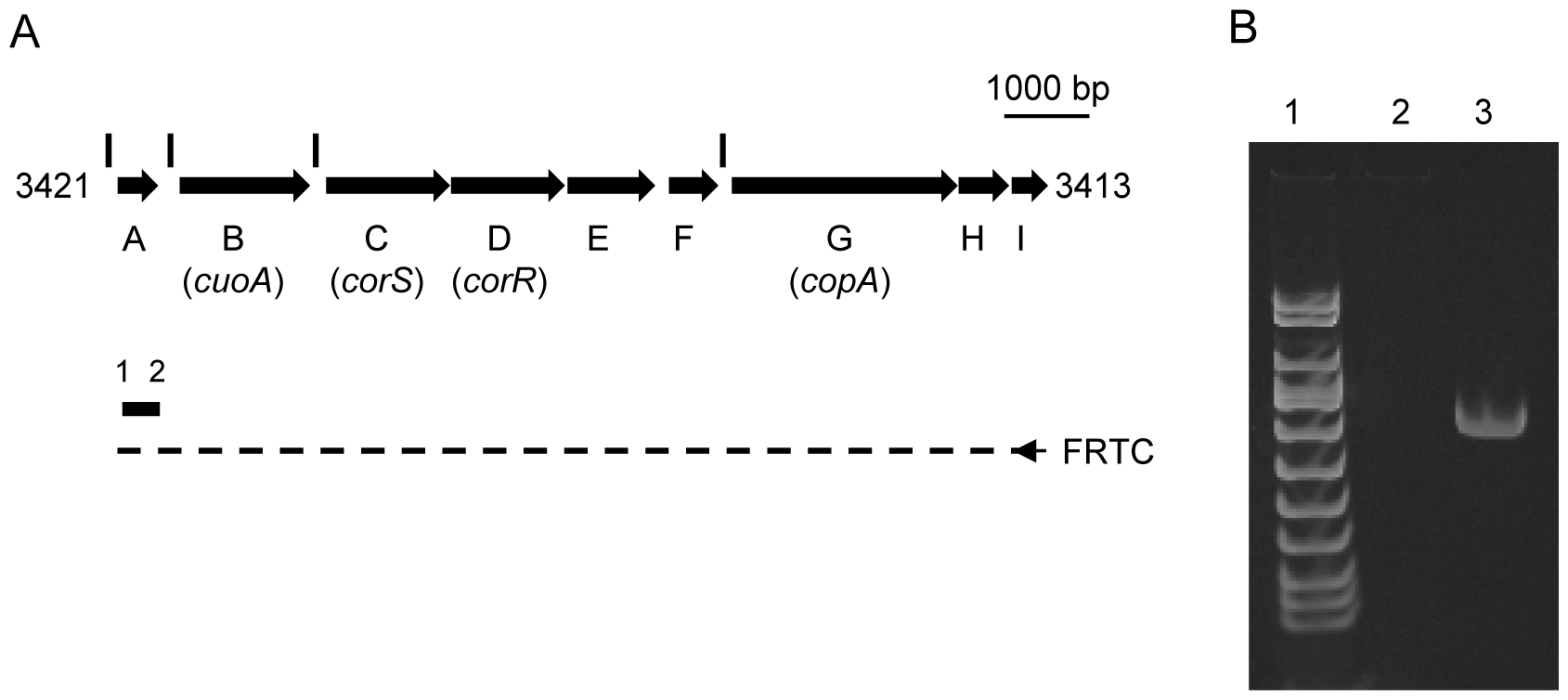
The *curA* operon includes nine genes. **A.** Strategy used to study the co-expression of the nine genes. cDNA is drawn as dashed line, with the region corresponding to the primer used to synthesize the cDNA represented by an arrow (not to scale). The name of the primer used to synthesize the cDNA is indicated on the left. PCR product is drawn as a solid line above the cDNA used as a template. Primers used for PCR are indicated with numbers, 1 corresponds to ORTF, and 2 corresponds to ORTR. Sequences of all primers are shown in Table S1. Vertical lines upstream of genes A, B, C, and G indicate the position of 4 putative σ^54^ promoters (see Fig. S2 for details). **B.** RT-PCR experiment. Lane 1 contains DNA molecular weight marker VIII (Roche). Lane 2, negative control using total RNA as a template. Lane 3, PCR product obtained using cDNA as a template (see panel A).

Genes encoding the MCO CuoA (MXAN_3420) and the P_1B_-type ATPase CopA (MXAN_3415) are both up-regulated by copper addition and exhibit similar expression profiles [6], [8]. They are located in the copper region 2 of the *M. xanthus* chromosome where nine genes are encoded in the same orientation (Fig. 1A, gene identifiers range from MXAN_3413 to 3421). Co-expression of these genes was examined using total RNA from vegetative cells grown with 300 µM copper. This RNA was used as a template for RT-PCR (reverse-transcription PCR) synthesis of cDNA as depicted in Fig. 1A. PCR was performed using this cDNA or total RNA as templates and the oligonucleotides pairs ORTR and ORTF as primers (Table S1). The PCR reaction from the cDNA template amplified one fragment with the expected size (lane 3 in Fig. 1B). A reaction in which no cDNA was previously synthesized from the RNA sample yielded no PCR product (lane 2 in Fig. 1B) indicating minimal DNA contamination of the RNA prep. This result suggests that these nine genes form an operon, which has been designated *curA* (for Cu resistance).

**Figure 2:**
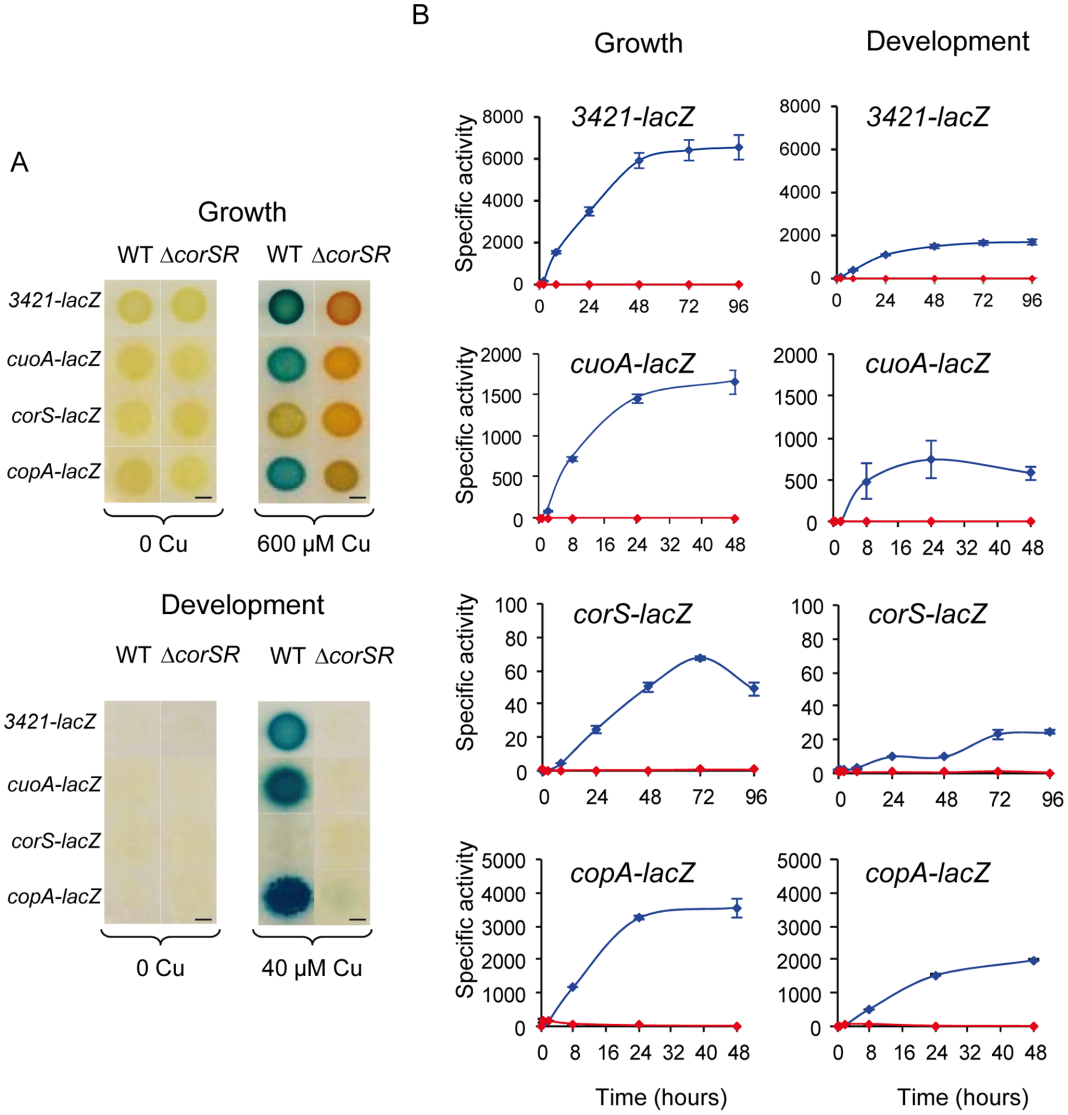
Genes regulated by CorSR. **A.** Qualitative analysis of the CorSR regulated genes. Cells of the different strains were inoculated on rich (growth) or starvation (development) media containing X-gal. Pictures were taken after 48 h of incubation. Bars, 3 mm. **B.** Quantitative analysis of the expression of *MXAN_3421*, *cuoA*, *corS,* and *copA* in the WT strain and the Δ*corSR* mutant. Plasmids containing *MXAN_3421-lacZ*, *cuoA-lacZ*, *corS-lacZ*, and *copA-lacZ* were introduced into WT (blue lines) or Δ*corSR* (red lines) backgrounds, and incubated for growth conditions on CTT agar plates containing 600 µM copper. For development, cells were incubated on CF agar plates containing 40 µM copper. Specific β-galactosidase activity in the cell extracts of all the strains was determined as described in Materials and Methods. Note the difference in the scales between panels. Error bars indicate standard deviations.

To further verify that *curA* expression is up-regulated by copper, as was previously demonstrated for two genes of the operon, *cuoA* and *copA* [6], [8], a strain harboring a fusion of the start codon of gene *MXAN_3421* with *lacZ* was generated. As shown in Fig. 2, copper is required for expression. Copper up-regulates expression with a plateau between 48 and 72 h after copper addition during growth and 24 and 48 h during development. This expression profiles are similar to those reported for *cuoA* and *copA*, corroborating that the nine genes form an operon.

The predicted locations and functions of the proteins encoded by the nine genes are shown in Table 1. These predicted functions were inferred from BLASTP similarity searches and scanning for protein families (Pfam) at the Sanger Institute [10]. Domains were determined by using ScanProsite, MotifScan, and InterProScan at ExPASy server (http://expasy.org/tools/). Along with the previously studied genes *cuoA* and *copA* [6], [8], the cluster includes two genes encoding a TCS, which has been termed CorS (for copper resistance sensor histidine kinase) and CorR (for copper resistance response regulator). Except for the response regulator CorR, all the proteins encoded in the operon are predicted to be located in the cell envelope (Table 1).

**Table 1:** *In silico* analysis of the nine genes of the *curA* operon.

MXAN^a^	Protein	Protein Predicted Function^b^	SP^c^/TM^d^
3421	A	Protein with His-rich region, cupredoxin1(PF13473)	Yes/No
3420	B (CuoA)	Multicopper oxidase (PF07732, PF07731, PF00394)	Yes/No
3419	C (CorS)	Signal transduction histidine kinase (PF02518, PF00512, PF13581, PF00672, PF13589)	Yes/Yes (1)
3418	D (CorR)	σ^54^-dependent DNA-binding response regulator (PF00072, PF00158, PF02954)	No/No
3417	E	Lipoprotein containing two PQQ_2 domains (PF13360)	Yes/No
3416	F	Lipoprotein containing DUF305 domain (PF03713)	Yes/No
3415	G (CopA)	Copper-translocating P-type ATPase (PF04945, PF00122, PF02954)	No/Yes (7)
3414	H	Cytochrome *c* oxidase, cbb3-type, subunit III (PF13442)	Yes/No
3413	I	Hypothetical protein	Yes/No

a MXAN: gene identifiers in *M. xanthus* genome.

b Predicted function inferred from BLASTP, Pfam, ScanProsite, MotifScan, and InterProScan. The Pfam identifiers are in parentheses.

c Signal peptides (SP) were deduced from results at SignalP and SOSUI servers.

d Transmembrane domains (TM) from TMHMM and SOSUI servers. The numbers of predicted transmembrane domains are indicated in parentheses.

Programs and databases used are available through ExPASy server (http://www.expasy.org/).

### The Copper-responsive *curA* Operon is under Control of the Two-component System CorSR

Three genes of the *curA* operon, *MXAN_3421* (this study), *cuoA* and *copA*, are known to be up-regulated by copper [6], [8]. They exhibit an expression plateau after metal supplementation. Attention was focused on the TCS CorSR as a possible regulator. The histidine kinase CorS was predicted to contain a signal peptide and one transmembrane domain, indicating a topology where the sensor domain was located in the periplasm, while the HAMP and the histidine kinase domains face the cytoplasm (Fig. S1). The response regulator CorR contains a σ^54^ interaction domain and, therefore, is predicted to function as a bacterial enhancer-binding protein to activate expression of genes from promoters recognized by this σ factor (Fig. S1). A bioinformatics search with the PromScan@Queen’s program (http://molbiol-tools.ca/promscan/) and Virtual Footprint software (http://prodoric.tu-bs.de/vfp/index2.php) to localize the putative σ^54^ promoters in the *curA* operon revealed the presence of four sequences with similarities to those reported to be recognized by this σ factor (Fig. S2).

An in-frame deletion (Δ*corSR*) was constructed. This mutant and the wild-type (WT) strain were used as genetic backgrounds to introduce the transcriptional fusions *MXAN_3421* (this study), *cuoA-lacZ* [6], *copA-lacZ* [8], and *corS-lacZ* (this study). As shown in Fig. 2, CorSR up-regulates *MXAN_3421*, *cuoA* and *copA* during growth and development in the presence of copper. Although *corS* expression follows a profile similar to those of the other three genes in the WT strain (Fig. 2B), it should be noted that specific β-galactosidase activity levels reached in the presence of copper were significantly lower for this gene and that maxima were reached later.

**Figure 3:**
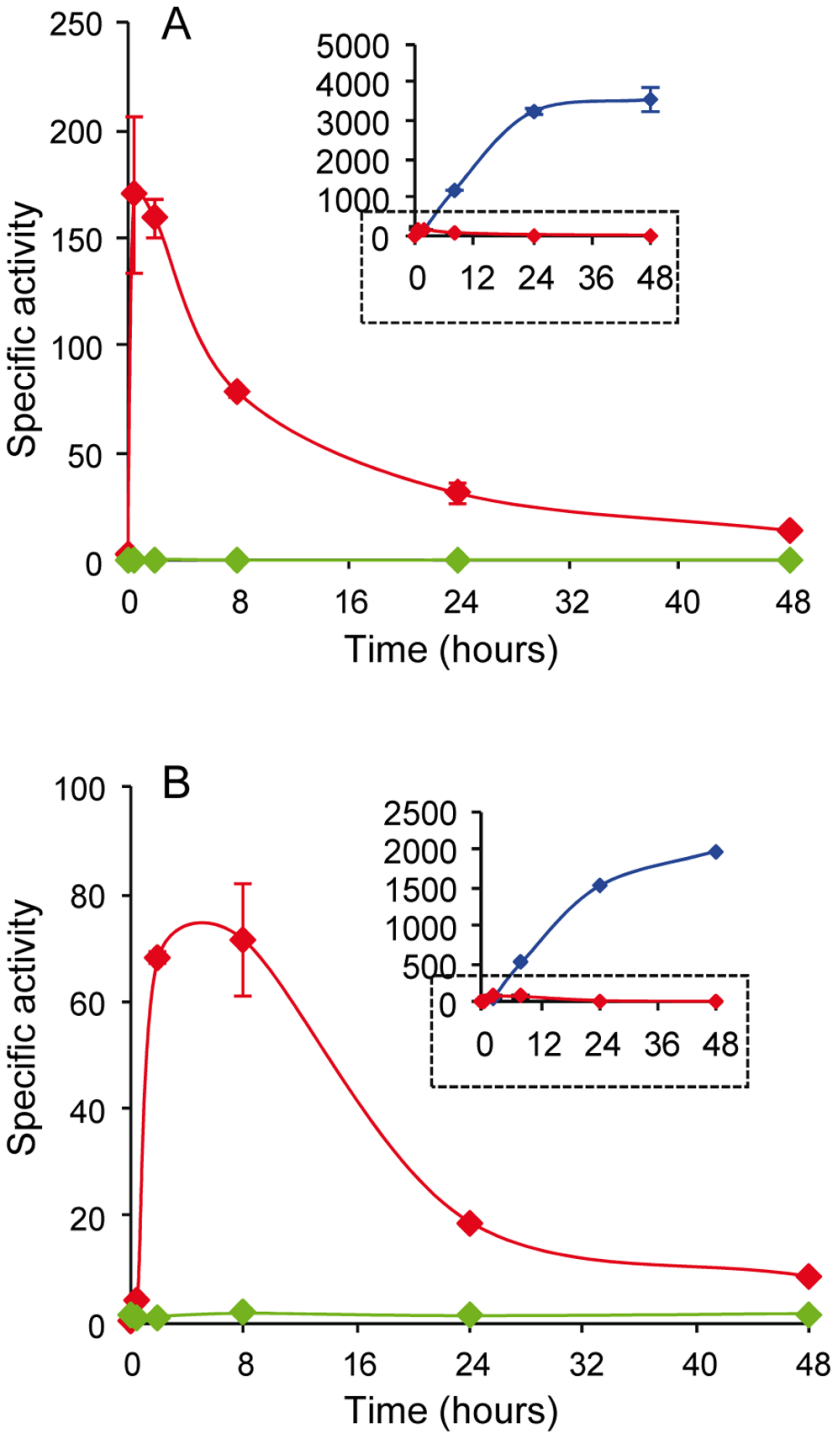
The expression of *copA* is regulated by CorSR and the ECF σ factor CorE. Plasmids containing *copA-lacZ* fusion were introduced into three different genetic backgrounds: WT (blue lines), Δ*corSR* (red lines), and the double mutant Δ*corSR* Δ*corE* (green lines). **A.** Cells were incubated on CTT agar plates (growth) containing 600 µM copper. **B.** Cells were incubated on CF agar plates (development) containing 40 µM copper. Small figures show the expression profiles of *copA* in the WT and Δ*corSR* backgrounds represented in Fig. 2. Note the difference in the scale between small and large graphics. Error bars indicate standard deviations.

While deletion of the TCS reduced most of the copper-dependent expression of the P_1B_-type ATPase *copA*, some up-regulation still remained (Fig. 2A) that is not evident in the plots shown in Fig. 2B. The up-regulation is observed at a finer scale in Fig. 3, with an expression profile exhibiting a peak after copper addition. This expression profile is typical of those genes regulated by the ECF σ factor CorE [9]. Previous bioinformatics analyses identified a CorE-like promoter upstream of *copA*, and showed that the early expression of *copA* diminished in a Δ*corE* mutant [9]. A Δ*corSR* Δ*corE* mutant was constructed and the *copA-lacZ* fusion was introduced in this mutant. As shown in Fig. 3, *copA* expression was completely abolished in this double mutant during growth and development. These results demonstrate that *copA* is under control of two promoters. One promoter responds to CorE, showing a profile in which transcription peaks after copper addition, while the other responds to CorR-σ^54^, giving a profile in which the expression slowly increases in a time-dependent manner, reaching a plateau at 24 h.

Different expression levels of genes of the *curA* operon, identification of four putative σ^54^ promoters and the double regulation of *copA* indicate a complex regulation of this operon, where σ factors of the families σ^54^ and σ^70^ participate.

### Phenotype of the Δ*corSR* Mutant Strain during Growth and Development

**Figure 4:**
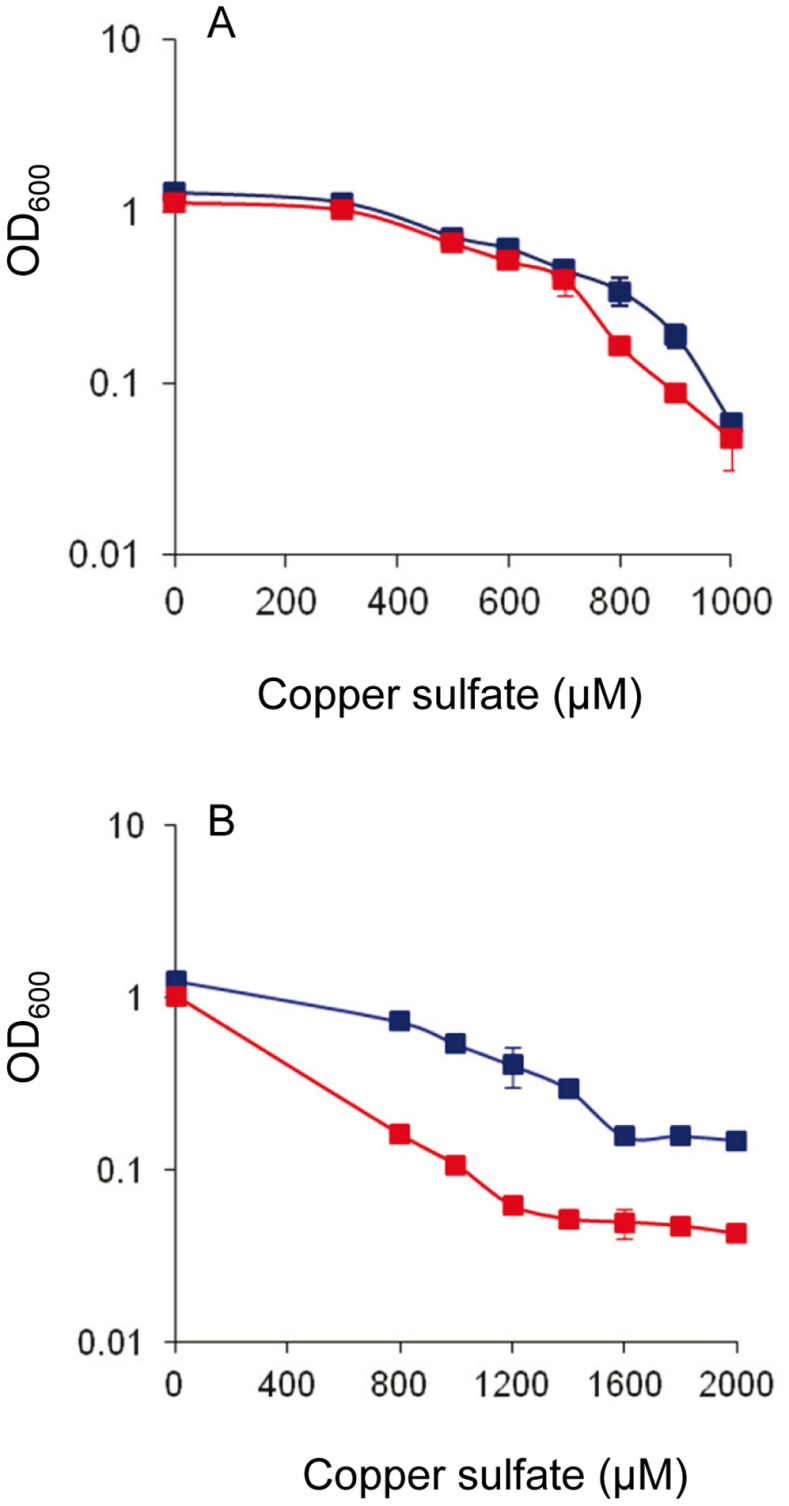
Growth of WT and Δ*corSR* strains in the presence of copper. **A.** Cells grown in the absence of copper were diluted into CTT liquid media containing the indicated copper concentrations. **B.** Cells were adapted to grow in the presence of 600 µM of copper prior to dilution in CTT liquid media containing the indicated copper concentrations. OD_600_ in both panels was monitored after 24-h incubation. Error bars indicate standard deviations. WT, blue lines; Δ*corSR,* red lines.

The phenotype of the Δ*corSR* mutant was analyzed. First, growth in the presence of different copper concentrations was determined. As shown in Fig. 4A, cell yields in the presence of several copper concentrations were only slightly lower than those obtained with the WT strain. In contrast, when cells were previously adapted to live in the presence of 600 µM copper, the Δ*corSR* mutant was more sensitive (Fig. 4B). This result corroborates a role of this system in copper detoxification.

**Figure 5:**
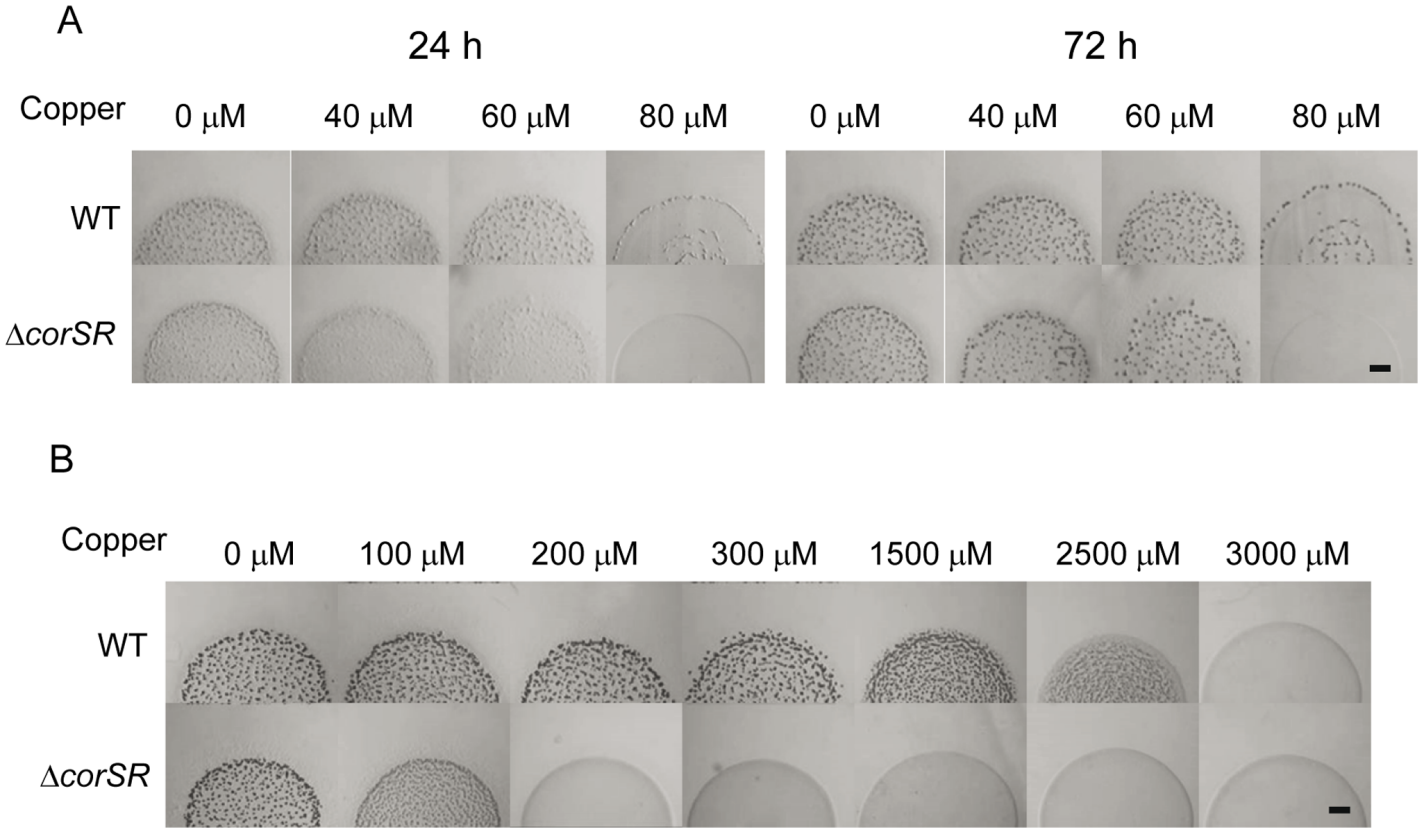
Development of the WT strain and the Δ*corSR* mutant in the presence of copper. **A.** Cells grown in the absence of copper were concentrated at an OD_600_ of 15 and spotted onto CF medium with the copper concentrations indicated. **B.** Cells grown in the presence of 600 µM copper sulfate were spotted onto CF agar plates with the indicated metal concentrations. Photographs in this panel were taken at 72 h of incubation. Bars, 1 mm.

On starvation media, non-pre-adapted Δ*corSR* cells were impaired in development (Fig. 5A) even at low copper concentrations. However, the difference in copper resistance between the WT strain and the mutant was more dramatic when development was assayed with pre-adapted cells. In this case, the mutant was able to form fruiting bodies only up to 100 µM copper (Fig. 5B), while the WT aggregated up to 2500 µM. Nevertheless, although the mutant strain was unable to form fruiting bodies, cells remained viable up to 2000 µM, as deduced after streaking cells from these spots on rich media. These results indicate that copper inhibits the normal developmental program in the mutant.

**Figure 6:**
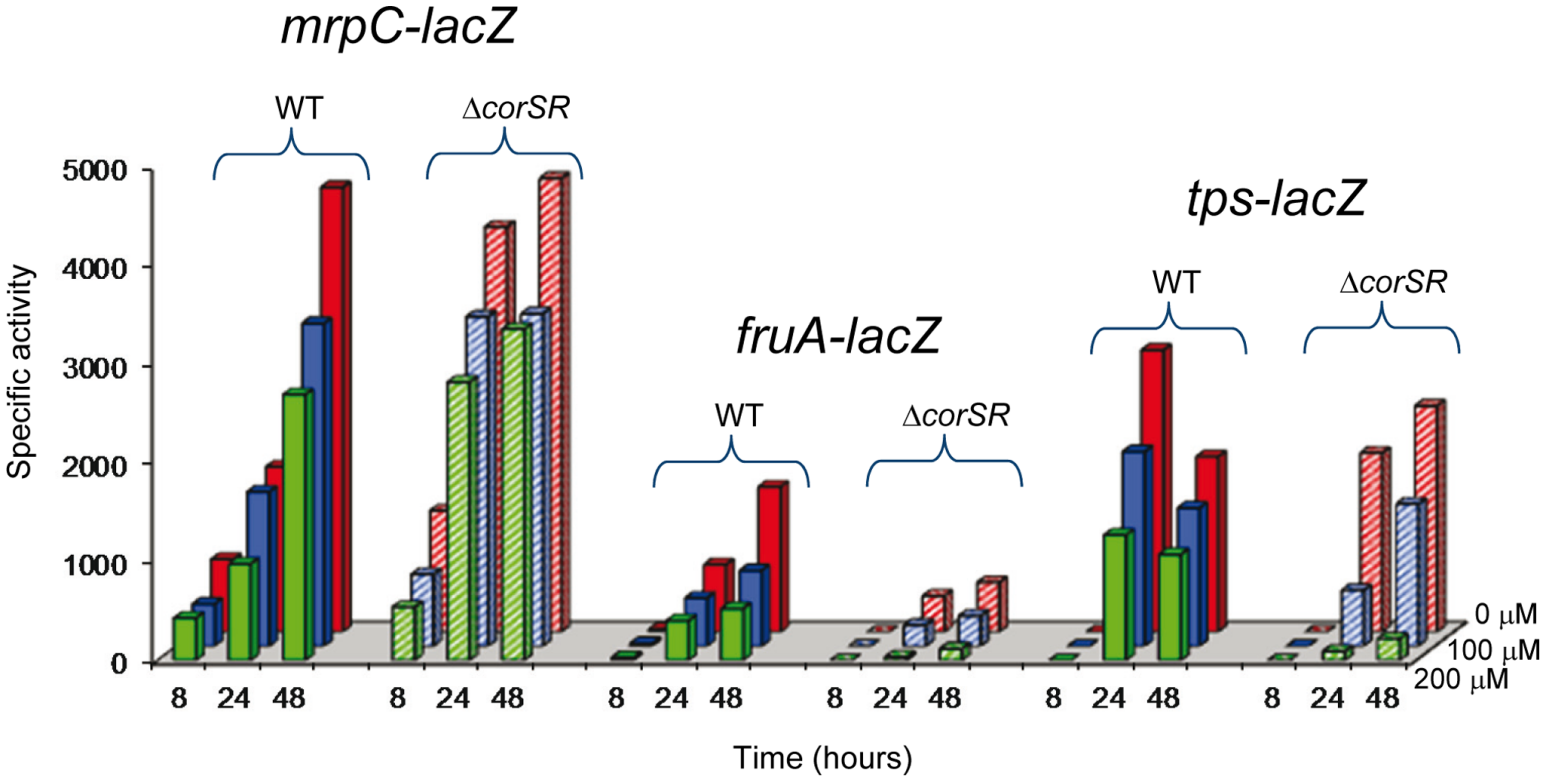
Expression of three developmental genes, *mrpC*, *fruA* and *tps*, in copper pre-adapted cells. WT (solid columns) or Δ*corSR* (dashed columns) cells harboring *mrpC-lacZ*, *fruA-lacZ* and *tps-lacZ* were grown in the presence of 600 µM copper, concentrated to an OD_600_ of 15, and spotted onto CF medium containing the indicated copper concentrations. Cells were harvested at several time-points to determine specific β-galactosidase activity.

In order to elucidate why copper pre-adapted mutant cells were unable to aggregate, we examined expression of three developmental genes that are sequentially expressed: *mrpC*, which encodes a transcriptional regulator that regulates the expression of *fruA* [11]; *fruA*, which encodes a response regulator [12], [13]; and *tps*, which encodes protein S, the major spore coat protein, whose expression depends on FruA [14], [15]. Plasmids harboring *mrpC-lacZ*, *fruA-lacZ*, and *tps*-*lacZ* fusions were introduced into the WT and Δ*corSR* strains. Measurements of β-galactosidase activities were carried out under developmental conditions (CF media) with copper pre-adapted cells. As shown in Fig. 6, *mrpC* expression levels were slightly higher in the mutant than in the WT strain, even when cells were spotted onto starvation medium containing 200 µM copper (Fig. 6), a copper concentration that blocks fruiting body formation in the mutant (Fig. 5B). This result indicates that reduced *mrpC* expression is not responsible for inhibiting aggregation in the presence of metal. However, expression of *fruA* was clearly reduced in the Δ*corSR* mutant (Fig. 6). The timing of *tps* induction either in the absence or in the presence of copper was altered, although maximal expression levels are only slightly lower in the mutant than in the WT with 0 and 100 µM copper. Conversely, expression levels decreased drastically in the mutant when CF media contained 200 µM copper (Fig. 6). No fruiting bodies are produced by the mutant at this copper concentration (Fig. 5B). Altogether these data indicate that copper affects development of the Δ*corSR* mutant at a time-point between *mrpC* and *fruA* induction.

### Deletion of *MXAN_3414* Induces Carotenoid Biosynthesis at Low Copper Concentrations during Growth and Development

**Figure 7:**
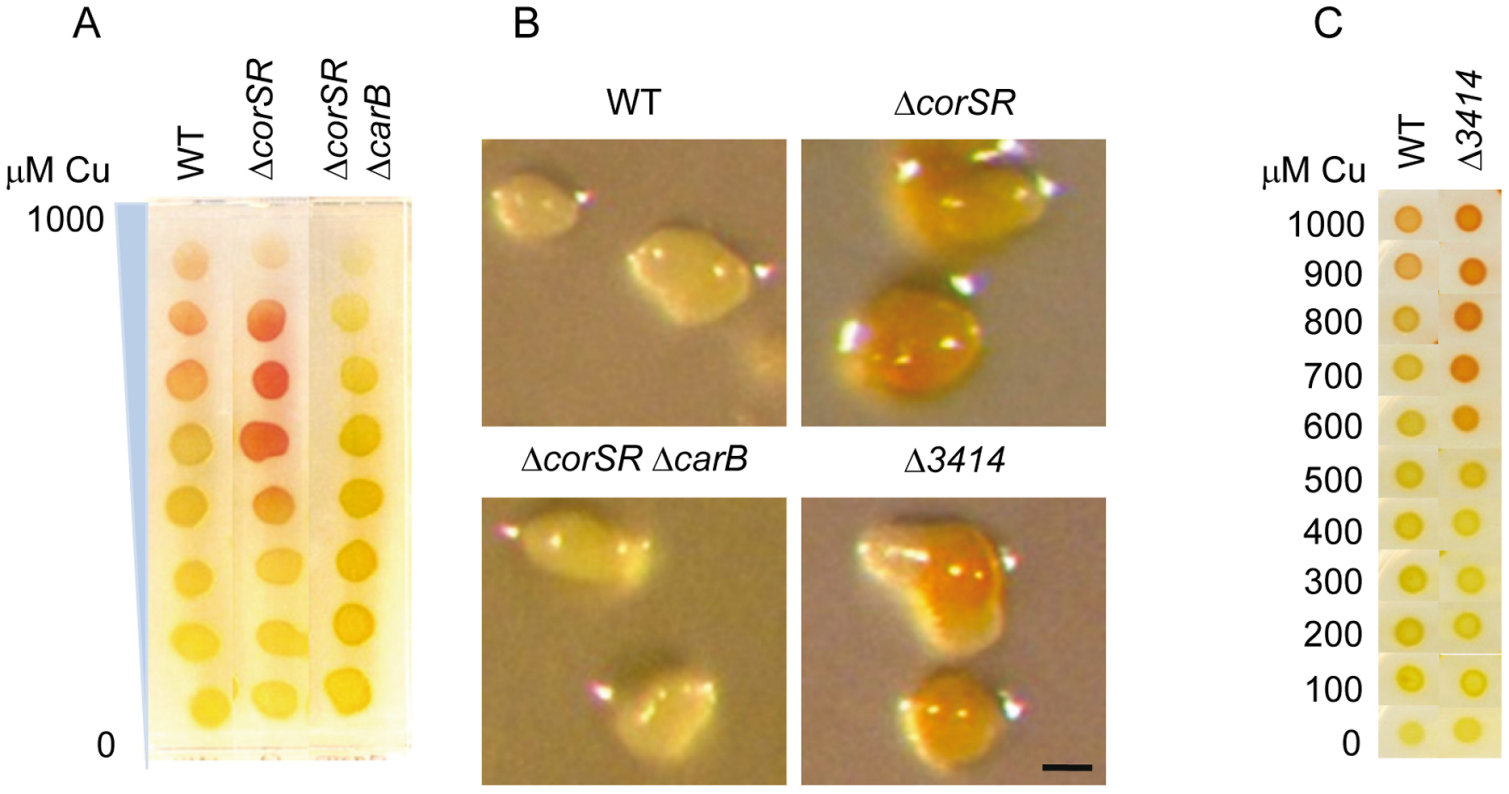
Carotenoid accumulation in different strains during growth and development. **A.** WT, Δ*corSR*, and Δ*corSR* Δ*carB* strains were grown in the absence of copper, concentrated at an OD_600_ of 15, and spotted onto CTT agar plates containing a copper gradient from 0 to 1000 µM. Pictures were taken after 48-h incubation. **B.** Carotenoid accumulation during development. In these experiments cells grown in the absence of copper were concentrated at an OD_600_ of 15 and spotted onto CF media with 40 µM copper. Pictures were taken at 72 h. Bar represents 100 µm. **C.** Carotenoid accumulation in the Δ*3414* and the WT strains during growth in the presence of different copper concentrations. Cells were grown in the absence of copper, concentrated to an OD_600_ of 15, and spotted onto CTT agar plates containing the indicated copper concentrations.

When the Δ*corSR* mutant was grown in the presence of copper, cells turned red at lower copper concentrations than WT cells (Fig. 7A). During development (Fig. 7B), red fruiting bodies of the mutant were obtained with just 40 µM copper. In contrast, the WT strain did not turn red even with 80 µM copper, the highest concentration that can be used with non-preadapted cells during development [6]. To verify that the red color was due to accumulation of carotenoids, a mutant was constructed carrying a deletion in both the TCS *corSR* and the *carB* operon, which encodes all but one of the structural genes involved in carotenoid biosynthesis [16]. As shown in Fig. 7A and B, cells lacking *carB* did not turn red, indicating that the red color is due to carotenoid accumulation. This result indicates that deletion of *corSR* induces carotenoid biosynthesis by copper in the presence of low external metal availability.

Carotenoid-overproduction at low copper concentrations during growth and within fruiting bodies was not observed in the WT, or in single, double or triple deletion mutants for the copper homeostasis genes in *M. xanthus* [6]–[8]. In order to find the *corSR*-regulated gene involved in this phenotype, we analyzed the sequences and microsynteny conservation of the non-previously characterized genes of the *curA* cluster. The *in silico* search revealed that *MXAN_3414* encodes a protein that belongs to Pfam family PF13442, termed “cytochrome *c* oxidase (Cox), *cbb3*-type, subunit III”, with an E-value of 3.1e-10 (Table 1). *cbb3*-type cytochrome *c* oxidases (*cbb3*-Cox) are multisubunit metalloenzymes whose assembly and activity require the presence of copper in their catalytic subunits and are members of the cytochrome *c* oxidase superfamily [17]. *cbb3*-Cox are important for aerobic respiration, the onset of photosynthesis, rhizobial symbiosis, and pathogenesis in various species [18]. MXAN_3414 shows homologies and belongs to the same Pfam family as the well characterized FixP from *Bradyrhizobium japonicum* and CcoP from *Rhodobacter capsulatus* [19], [20]. However, *MXAN_3414* is not genetically linked to the rest of the Cox subunits, suggesting an alternative role for this protein. In fact, the *M. xanthus* genome encodes two paralogs of MXAN_3414, MXAN_5539 (17% identity, 56% similarity) and MXAN_5554 (19% identity; 54% similarity). Both of them may form clusters with different Cox subunits, indicating that they are the housekeeping Cox. Analysis of the microsynteny of the 50 best-fit sequences showed that 28 orthologous genes of MXAN_3414 appeared genetically linked to genes encoding proteins involved in a copper response (Fig. S3). The alignments of these 29 proteins and the representative domain architecture are indicated in Fig. S4.

The results of these analyses make MXAN_3414 the leading candidate for carotenoid induction observed in the Δ*corSR* mutant. As part of a cytochrome *c* oxidase up-regulated by copper, MXAN_3414 may generate energy in the presence of this metal. Accordingly, cells where this gene is not up-regulated (as in the case of the Δ*corSR* mutant) would be deficient in energy generation when cultured with copper. As mentioned in the Introduction, carotenoid biosynthesis induced by copper is more efficient under sub-optimal growth conditions [5], so it is expected that Δ*corSR* would be subjected to sub-optimal growth conditions in the presence of copper due to the lack of MXAN_3414. To examine this hypothesis, we constructed and analyzed an in-frame deletion mutant of *MXAN_3414*. The copper-induced carotenogenesis profiles of the Δ*3414* strain were similar to those of the Δ*corSR* mutant during growth and development (Fig. 7B and C), indicating that lack of *MXAN_3414* expression is responsible for carotenoid accumulation in the Δ*corSR* mutant.

**Figure 8:**
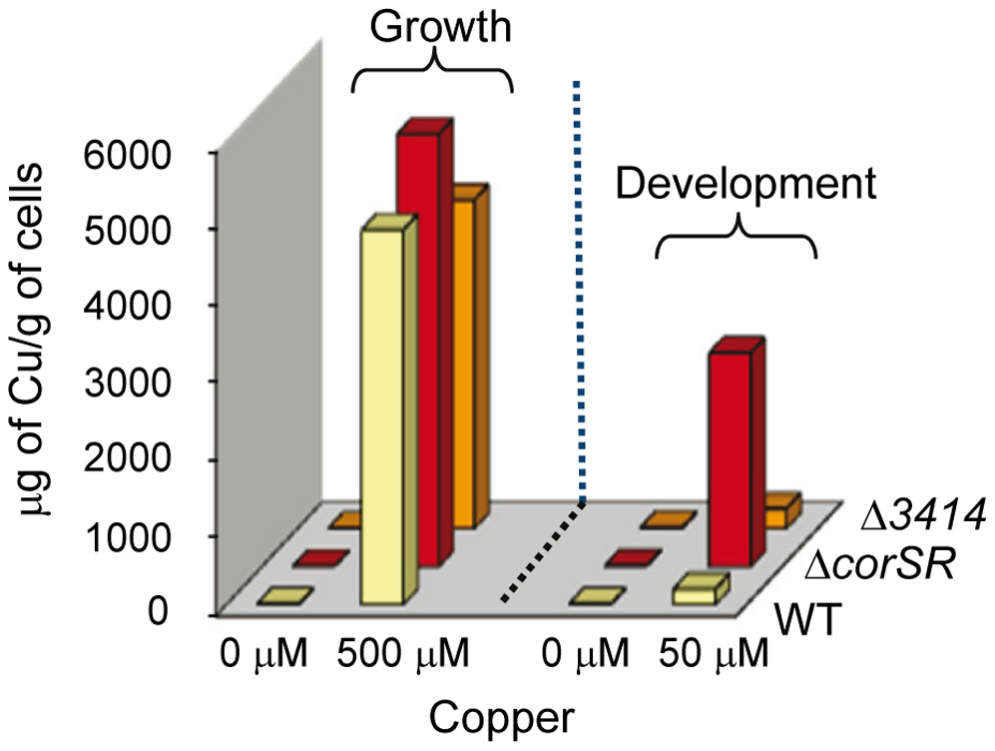
Measurements of intracellular copper accumulation during growth and development. Cells from WT, Δ*corSR,* and Δ*3414* strains were incubated on CTT (growth) or CF (development) agar plates containing the indicated copper concentrations for 72 h prior to harvesting for copper determinations.

Finally, we decided to test whether carotenoid induction could be related to copper accumulation. Determination of the intracellular copper accumulated by the WT strain and the Δ*corSR* and Δ*3414* mutants revealed that the Δ*corSR* mutant accumulates more copper than the WT strain, 1.3-fold during growth and 13-fold during development (Fig. 8). However, the Δ*3414* mutant accumulated the same amount of metal as the WT (Fig. 8) while producing more carotenoids, so carotenoid biogenesis is not strictly related to copper accumulation.

## Discussion

The large number of genes involved in copper detoxification mechanisms in *M. xanthus* and their differential regulation suggests that several transcriptional regulators may be involved. Only the copper-dependent ECF σ factor CorE is known to induce expression of those genes involved in rapid adaptation [9]. Here we report the identification of the second regulatory system consisting of the histidine kinase CorS and the response regulator CorR. This TCS regulates expression of genes that exhibit a slow up-regulation after copper addition and reach a plateau after 24–48 h incubation with the metal. CorSR regulates the *curA* operon, which consists of nine genes, including genes for the MCO CuoA, the ATPase CopA, and the TCS CorSR.

Two lines of evidence indicate that regulation of the *curA* operon is complex. First, expression of genes located at different positions in the operon differs in the levels obtained for each one as well as the timing for when the plateau is reached. Second, *copA* is not only regulated by CorSR, but also by CorE. Accordingly, four different putative σ^54^-dependent promoters have been identified along the *curA* operon in this study and one putative CorE promoter upstream of *copA* was previously identified [9]. As three paralogous genes encoding P_1B_-type ATPases (*copA*, *copB,* and *copC*) have been identified in the *M. xanthus* genome [8], *copA* double regulation suggests that both the structural gene and promoter were duplicated. According to Teichmann and Babu [21], divergence after duplication of a target gene can lead to the duplicated gene remaining under the control of the same transcription factor or coming under the control of a different transcription factor. In our case, the data suggest that during the duplication event the promoter for the ancestral transcription factor has been maintained and a promoter for a new transcription factor has been gained.

Phenotypic analyses of the Δ*corSR* mutant have revealed that the *curA* cluster is involved in copper detoxification during growth and development. However, the fact that the mutant cells accumulated 13-fold more copper than the WT strain during development, but only 1.3-fold during growth, and that the effect on fruiting body formation is more drastic than on growth in the mutant in the presence of copper indicate that this TCS, and so that the *curA* cluster, is especially important for copper detoxification of developing cells.

We assayed expression of three sequential developmental genes (*mprC*, *fruA,* and *tps*) in the WT and mutant in the presence and absence of copper. The results indicate that *mprC* expression is not reduced in the Δ*corSR* mutant. Conversely, *fruA* and *tps* expressions are drastically reduced in the TCS mutant, especially in the presence of 200 µM of copper. Development at this copper concentration is blocked in the mutant, although cells are viable. As the active form of MrpC is the proteolytic product MrpC2 [22], a feasible explanation could be that *mprC* is expressed in the Δ*corSR* mutant but not properly processed in the presence of copper, reducing the expression of *fruA* (which depends on MrpC2) and *tps* (which depends on FruA). The protease responsible for the proteolytic cleavage of MrpC has not been identified yet, although it has been proposed that LonD could be responsible for this activity [22]. Processing of MrpC in the presence of copper and inhibition of the protease activity by this metal remain to be elucidated.

Another interesting observation is that CorSR prevents the formation of carotenoids at low copper concentrations. As the phenotype observed in the Δ*corSR* mutant is not observed in other mutants generated in genes of the *curA* operon, such as Δ*copA* or Δ*cuoA* [6], [8], we decided to investigate which gene is responsible for this effect. Herein, we demonstrate that the major contributor to the carotenoid phenotype of Δ*corSR* at low copper concentrations is the protein encoded by the gene *MXAN_3414*. The reason why this mutant induces carotenoid biosynthesis is not clear. However, it can be ruled out that it is an effect of intracellular copper accumulation, because mutant Δ*3414* accumulates similar amounts of copper to the WT strain, but turns red at low external copper concentrations during growth and forms red fruiting bodies. We reported several years ago that the copper induction of carotenoid synthesis in *M. xanthus* is more effective at suboptimal growth conditions [5]. One plausible explanation for the phenotype of the Δ*3414* mutant could be that the protein encoded by this gene is required for energy production upon copper addition. The absence of this protein in the Δ*3414* mutant could reduce energy levels, mimicking growth in suboptimal conditions, and inducing carotenogenesis to lower copper concentrations. This explanation is supported by the homology of MXAN_3414 with the subunit III of *cbb3*-Cox. A linkage between copper resistance, Cox biogenesis, and competitive fitness has previously been reported in *Pseudomonas fluorescens* [23] and *Salmonella typhimurium* [24]. Our results reinforce this connection. Supporting the implication of MXAN_3414 and other members of PF13442 in copper tolerance is syntenic conservation of several orthologous of this cytochrome *c*-like family gene with genes coding for copper-response related proteins such as P_1B_-type ATPases, MCOs, or HRD heavy metal efflux systems (Fig. S3). Many bacterial species have several respiratory oxidases to allow cells to adapt their respiratory systems to meet the demands of a variety of environmental conditions, including different concentrations of metal ion cofactors [23]. It is tempting to speculate that MXAN_3414 could be an alternative Cox subunit that contributes to *M. xanthus* fitness in the presence of copper.

## Materials and Methods

### Bacterial Strains, Plasmids, and Growth Conditions

*M. xanthus* and *Escherichia coli* strains, oligonucleotides, and plasmids used in this study are listed in Tables S1 and S2. Luria-Bertani broth, supplemented with kanamycin (25 µg/ml) or X-gal (5-bromo-4-chloro-3-indolyl-β-D-galactopyranoside, 40 µg/ml) when needed, was used to grow *E. coli* strains at 37°C.

All *M. xanthus* strains were grown at 30°C with vigorous shaking (300 rpm) in CTT broth [25]. CTT agar plates contained 1.5% Bacto agar (Difco). When necessary, kanamycin (80 µg/ml), X-gal (100 µg/ml), or galactose (10 mg/ml) were added. When needed, CTT medium was supplemented with copper at the concentrations indicated in each figure. CTT agar plates containing copper gradient in a range of concentrations between 0 and 1000 µM were prepared according to Moraleda-Muñoz *et al*. [5].

Starvation medium CF [25] was used to induce development, which was supplemented with copper at the concentrations indicated in the figures when needed. Cells were grown in CTT broth to approximately 3.0×10^8^ cells/ml (optical density at 600 nm [OD_600_] of 1) and resuspended to 4.5×10^9^ cells/ml (OD_600_ of 15) in TM buffer (10 mM Tris-HCl [pH 7.6], 1 mM MgSO_4_). Ten microliters of each suspension were spotted onto CF agar plates and incubated at 30°C, with observation on a Wild Heerbrugg dissecting microscope.

### Nucleic Acid Manipulations

Routine molecular biology techniques were followed for nucleic acid manipulations [26]. Total RNA from cultures containing 300 µM copper was prepared with the High Pure RNA Isolation kit provided by Roche. Samples were treated with the kit DNA-free (Ambion) to completely remove chromosomal DNA. The RNA was then subjected to reverse transcription using the primer FRTC, which anneals in gene MXAN_3413 (see Table S1). PCR was carried out with primers ORTR and ORTF, which anneal inside gene *MXAN_3421* (Fig. 1 and Table S1) using as templates total RNA or cDNA synthesized using FRTC as primer.

### Construction of In-frame Deletion Mutants

In-frame deletion mutants have been constructed as previously reported [6]. Concisely, in order to generate the corresponding plasmids (Table S2), the desired fragments were amplified by PCR, using WT chromosomal DNA as a template, the appropriate oligonucleotide pairs (Table S1), and the high-fidelity polymerase PrimeSTARS (Takara). PCR products were ligated to vector pBJ113 [27] to obtain plasmids harboring deletions of the corresponding genes (Table S2). The resulting non-replicating plasmids carrying the deletions were introduced into *M. xanthus* cells by electroporation [28]. Chromosomal integration was selected by plating cells onto CTT plates containing kanamycin (positive selection). Several randomly chosen kanamycin-resistant (Km^R^) merodiploids were analyzed by Southern blot hybridization for the proper recombination event. One positive strain was then grown in the absence of kanamycin and plated onto CTT plates containing galactose for negative selection. Southern blot analysis was used to screen kanamycin-sensitive (Km^S^) and galactose-resistant (Gal^R^) colonies, searching for the loss of the WT allele.

### Construction and Assay of Strains Harboring *lacZ* Fusions

The *lacZ* fusion plasmids were constructed by using vector pKY481 [29]. Fragments surrounding the upstream genes regions were amplified from WT chromosomal DNA by the use of the oligonucleotide pairs specified in Table S1. The BamHI site in the primers was introduced at the start codon of the corresponding genes and in frame with the BamHI site existing in the *lacZ* gene of plasmid pKY481. PCR products were digested with KpnI-BamHI, XhoI-BamHI or PstI-BamH1, and ligated into vector pKY481 digested with the same enzymes. The resulting plasmids were introduced into *M. xanthus* (WT or mutants) cells by electroporation, and the Km^R^ colonies were analyzed by Southern blotting. Strains containing *lacZ* fusions were incubated at 30°C on CTT and CF agar plates containing different metal concentrations. For qualitative analyses of β-galactosidase activity, plates containing X-gal were used. For quantitative analyses, cell extracts were obtained at different times by sonication and assayed for activity as previously reported [5]. The amount of protein in the supernatants was determined by using the Bio-Rad protein assay with bovine serum albumin as a standard. Specific β-galactosidase activity is expressed as nmol of *o*-nitrophenol produced per min and mg of protein. Measurements shown are the averages of data from triplicate experiments.

### Copper Accumulation Measurements

For determinations of copper accumulation, cultures grown overnight in CTT medium were concentrated to an OD_600_ of 15 as described above. Ten drops of 20 µl each were spotted onto CTT agar plates supplemented with 0 or 500 µM copper (growth conditions), or onto CF agar plates supplemented with 0 or 50 µM copper (developmental conditions). After 72-h incubation at 30°C, cells were harvested into a 15-ml sterile polypropylene centrifuge tube and washed three times with modified CTT medium (without MgSO_4_ and containing 1 mM EDTA). Pellets were dried at 80°C overnight and dissolved in 5 ml of trace-metal-grade nitric acid by heating at 80°C for 30 min according to a method described previously by Outten et al. [30]. The metal content of the samples was measured by using Thermo Jarrell Ash Enviro 36 Inductively Coupled Plasma-Optical Emission Spectrograph with a 20 element sweep. The sample was nebulized and moved up into a torch with burning argon at over 1370°C. The light emitted from the burning of the sample was reflected back to a grating that spreads the light out like a prism, into the different wavelengths, which were picked up by photomultiplier tubes set at those wavelengths indicated beside the atomic symbol on the data sheet. The instrument was run by the EPA method 6010C.

## Supporting Information

Figure S1 **Domain architecture and location of the two-component system CorSR.** **A**. Domain distribution of the sensor histidine kinase CorS. SP, signal peptide (represented as an open box to indicate that it will not be a part of the mature protein); TM, transmembrane domain; HAMP, linker domain present in histidine kinases, adenyl cyclases, methyl-accepting proteins and phosphatases (PF00672), E-value 1.3e-15; His Kinase A (phosphoacceptor) domain (PF00512), E-value 1.1e-16, and HATPase_c domain (PF02518), E-value 7.5e-28, are components of histidine kinases. **B**. Domain distribution of the response regulator CorR. Response_reg: Response regulator receiver domain (PF00072), E-value 8.5e-29; Sigma54_activat, sigma-54 interaction domain (PF00158), E-value 1.9e-67; HTH_8, Bacterial regulatory protein, Fis family (PF02954), E-value 7.4e-05. OM, outer membrane; PS, periplasmic space; IM, inner membrane; C, cytoplasm.
(PDF)

Figure S2 ***In silico*** **analysis of the upstream sequences of the** ***curA*** **genes.** **A**. Four different σ^54^ promoters were found (green rectangles). **B**. Sequence alignments of the four putative promoters. Underlined nucleotides match the *E. coli* σ^54^ consensus: TGGCACGRNNNTTGCW described by Barrios *et al.* (1999). **C**. LOGO representation of the four putative promoters. **D**. LOGO obtained with the putative σ^54^ promoter sequences described in other *M. xanthus* genes (Kroos and Inouye, 2008).
(PDF)

Figure S3 **Microsynteny conservation in several bacteria in the genomic environment of genes coding for proteins with PF13442 domains (red arrows).** The other colored arrows represent genes with predicted function associated to copper response. Dark pink, Cu^2+^-exporting ATPase (PF00122, PF00702); light green, multicopper oxidase (PF07732, PF07731 and PF00394); dark green, copper resistance protein D, CopD (PF05425); yellow, copper resistance protein B, CopB (PF05275); orange, copper resistance protein C, CopC (PF04234); grey, copper binding periplasmic protein CusF (PF11604); purple, outer membrane efflux protein (PF02321); blue, heavy metal efflux system (PF02321, PF12700, PF 00529, PF00873); brown, two component regulatory system (PF02518, PF00512, PF00072).
(PDF)

Figure S4 **MXAN_3414-like proteins. A**. Alignments of the 29 proteins with the conserved PF13442 (cytochrome *c* oxidase, cbb3-type, subunit III) and genetic environments with copper related proteins represented in Figure S3. **B**. Representative domain architecture.
(PDF)

Table S1 **Oligonucleotides used in this study.**
(DOC)

Table S2 **Bacterial strains and plasmids used in this study.**
(DOC)
