# Supplementary material for: The Myxococcus xanthus Two-Component System CorSR Regulates Expression of a Gene Cluster Involved in Maintaining Copper Tolerance during Growth and Development
Source: PLoS One. 2013 Jul 10;8(7):e68240. doi: 10.1371/journal.pone.0068240 (PMC3707914; doi:10.1371/journal.pone.0068240)
Supplement: Figure S1 — Domain architecture and location of the two-component system CorSR. A. Domain distribution of the sensor histidine kinase CorS. SP, signal peptide (represented as an open box to indicate that it will not be a part of the mature protein); TM, transmembrane domain; HAMP, linker domain present in histidine kinases, adenyl cyclases, methyl-accepting proteins and phosphatases (PF00672), E-value 1.3e-15; His Kinase A (phosphoacceptor) domain (PF00512), E-value 1.1e-16, and HATPase_c domain (PF02518), E-value 7.5e-28, are components of histidine kinases. B. Domain distribution of the response regulator CorR. Response_reg: Response regulator receiver domain (PF00072), E-value 8.5e-29; Sigma54_activat, sigma-54 interaction domain (PF00158), E-value 1.9e-67; HTH_8, Bacterial regulatory protein, Fis family (PF02954), E-value 7.4e-05. OM, outer membrane; PS, periplasmic space; IM, inner membrane; C, cytoplasm. (PDF) [file pone.0068240.s001.pdf]

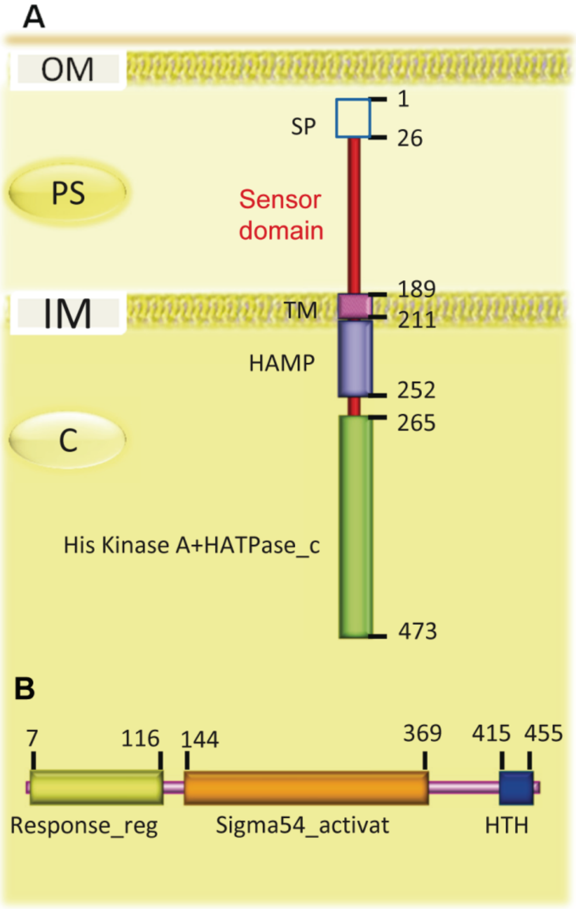

Figure S1. **Domain architecture and location of the two-component system CorSR**. **A**. Domain distribution of the sensor histidine kinase CorS. SP, signal peptide (represented as an open box to indicate that it will not be a part of the mature protein); TM, transmembrane domain; HAMP, linker domain present in histidine kinases, adenyl cyclases, methyl-accepting proteins and phosphatases (PF00672), E-value 1.3e-15; His Kinase A (phosphoacceptor) domain (PF00512), E-value 1.1e-16, and HATPase_c domain (PF02518), E-value 7.5e-28, are components of histidine kinases. **B**. Domain distribution of the response regulator CorR. Response_reg: Response regulator receiver domain (PF00072), E-value 8.5e-29; Sigma54_activat, sigma-54 interaction domain (PF00158), E-value 1.9e-67; HTH_8, Bacterial regulatory protein, Fis family (PF02954), E-value 7.4e-05. OM, outer membrane; PS, periplasmic space; IM, inner membrane; C, cytoplasm.
