# Supplementary material for: The Myxococcus xanthus Two-Component System CorSR Regulates Expression of a Gene Cluster Involved in Maintaining Copper Tolerance during Growth and Development
Source: PLoS One. 2013 Jul 10;8(7):e68240. doi: 10.1371/journal.pone.0068240 (PMC3707914; doi:10.1371/journal.pone.0068240)
Supplement: Figure S2 — In silico analysis of the upstream sequences of the curA genes. A. Four different σ54 promoters were found (green rectangles). B. Sequence alignments of the four putative promoters. Underlined nucleotides match the E. coli σ54 consensus: TGGCACGRNNNTTGCW described by Barrios et al. (1999). C. LOGO representation of the four putative promoters. D. LOGO obtained with the putative σ54 promoter sequences described in other M. xanthus genes (Kroos and Inouye, 2008). (PDF) [file pone.0068240.s002.pdf]

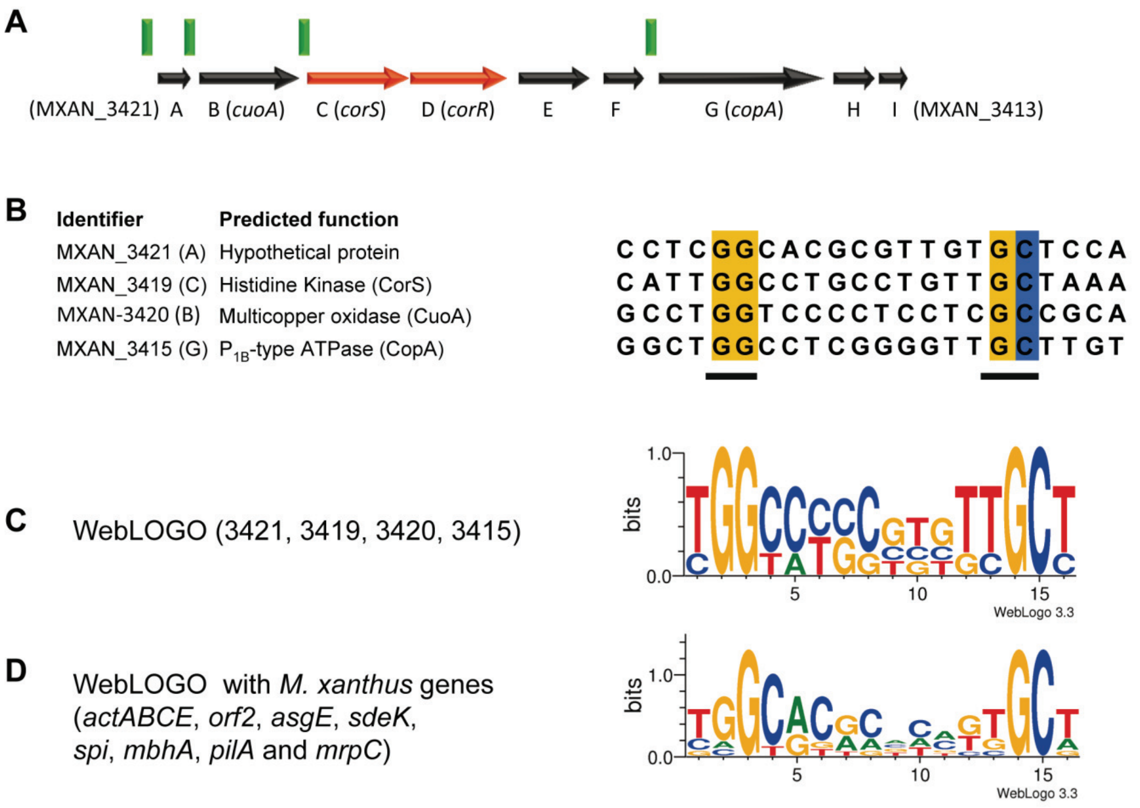

Figure S2. ***In silico*** **analysis of the upstream sequences of the** ***curA*** **genes**. **A**. Four different σ^{54} promoters were found (green rectangles). **B**. Sequence alignments of the four putative promoters. Underlined nucleotides match the *E. coli* σ^{54} consensus: TGGCACGRNNNTTGCW described by Barrios *et al.* (1999). **C**. LOGO representation of the four putative promoters. **D**. LOGO obtained with the putative σ^{54} promoter sequences described in other *M. xanthus* genes (Kroos and Inouye, 2008).

Barrios H, Valderrama B, Morett E (1999) Compilation and analysis of sigma(54)-dependent promoter sequences. Nucleic Acids Res.15: 4305-4313.

Kroos L, Inouye (2008) Transcriptional regulatory mechanisms during *Myxococcus xanthus* development. In Whitworth DE, editor. Myxobacteria. Multicelullarity and differentiation. Washington, DC, USA: ASM Press. pp. 149-168.
