# Supplementary material for: The Myxococcus xanthus Two-Component System CorSR Regulates Expression of a Gene Cluster Involved in Maintaining Copper Tolerance during Growth and Development
Source: PLoS One. 2013 Jul 10;8(7):e68240. doi: 10.1371/journal.pone.0068240 (PMC3707914; doi:10.1371/journal.pone.0068240)
Supplement: Figure S3 — Microsynteny conservation in several bacteria in the genomic environment of genes coding for proteins with PF13442 domains (red arrows). The other colored arrows represent genes with predicted function associated to copper response. Dark pink, Cu2+-exporting ATPase (PF00122, PF00702); light green, multicopper oxidase (PF07732, PF07731 and PF00394); dark green, copper resistance protein D, CopD (PF05425); yellow, copper resistance protein B, CopB (PF05275); orange, copper resistance protein C, CopC (PF04234); grey, copper binding periplasmic protein CusF (PF11604); purple, outer membrane efflux protein (PF02321); blue, heavy metal efflux system (PF02321, PF12700, PF 00529, PF00873); brown, two component regulatory system (PF02518, PF00512, PF00072). (PDF) [file pone.0068240.s003.pdf]

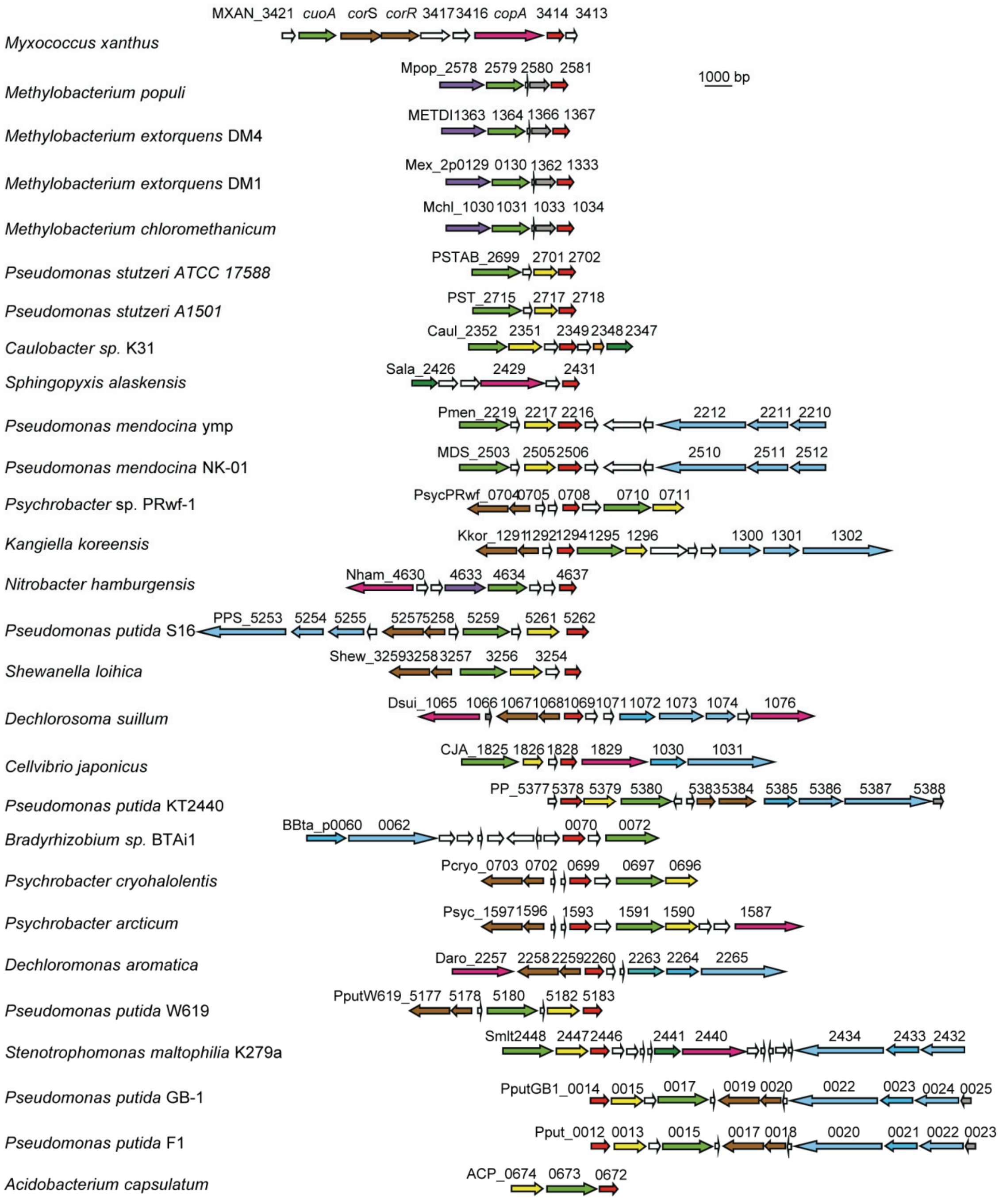

Figure S3. Microsynteny conservation in several bacteria in the genomic environment of genes coding for proteins with PF13442 domains (red arrows). The other colored arrows represent genes with predicted function associated to copper response. Dark pink, Cu^{2+}-exporting ATPase (PF00122, PF00702); light green, multicopper oxidase (PF07732, PF07731 and PF00394); dark green, copper resistance

protein D, CopD (PF05425); yellow, copper resistance protein B, CopB (PF05275); orange, copper resistance protein C, CopC (PF04234); grey, copper binding periplasmic protein CusF (PF11604); purple, outer membrane efflux protein (PF02321); blue, heavy metal efflux system (PF02321, PF12700, PF 00529, PF00873); brown, two component regulatory system (PF02518, PF00512, PF00072).
