# Supplementary material for: The Myxococcus xanthus Two-Component System CorSR Regulates Expression of a Gene Cluster Involved in Maintaining Copper Tolerance during Growth and Development
Source: PLoS One. 2013 Jul 10;8(7):e68240. doi: 10.1371/journal.pone.0068240 (PMC3707914; doi:10.1371/journal.pone.0068240)
Supplement: Figure S4 — MXAN_3414-like proteins. A. Alignments of the 29 proteins with the conserved PF13442 (cytochrome c oxidase, cbb3-type, subunit III) and genetic environments with copper related proteins represented in Figure S3. B. Representative domain architecture. (PDF) [file pone.0068240.s004.pdf]

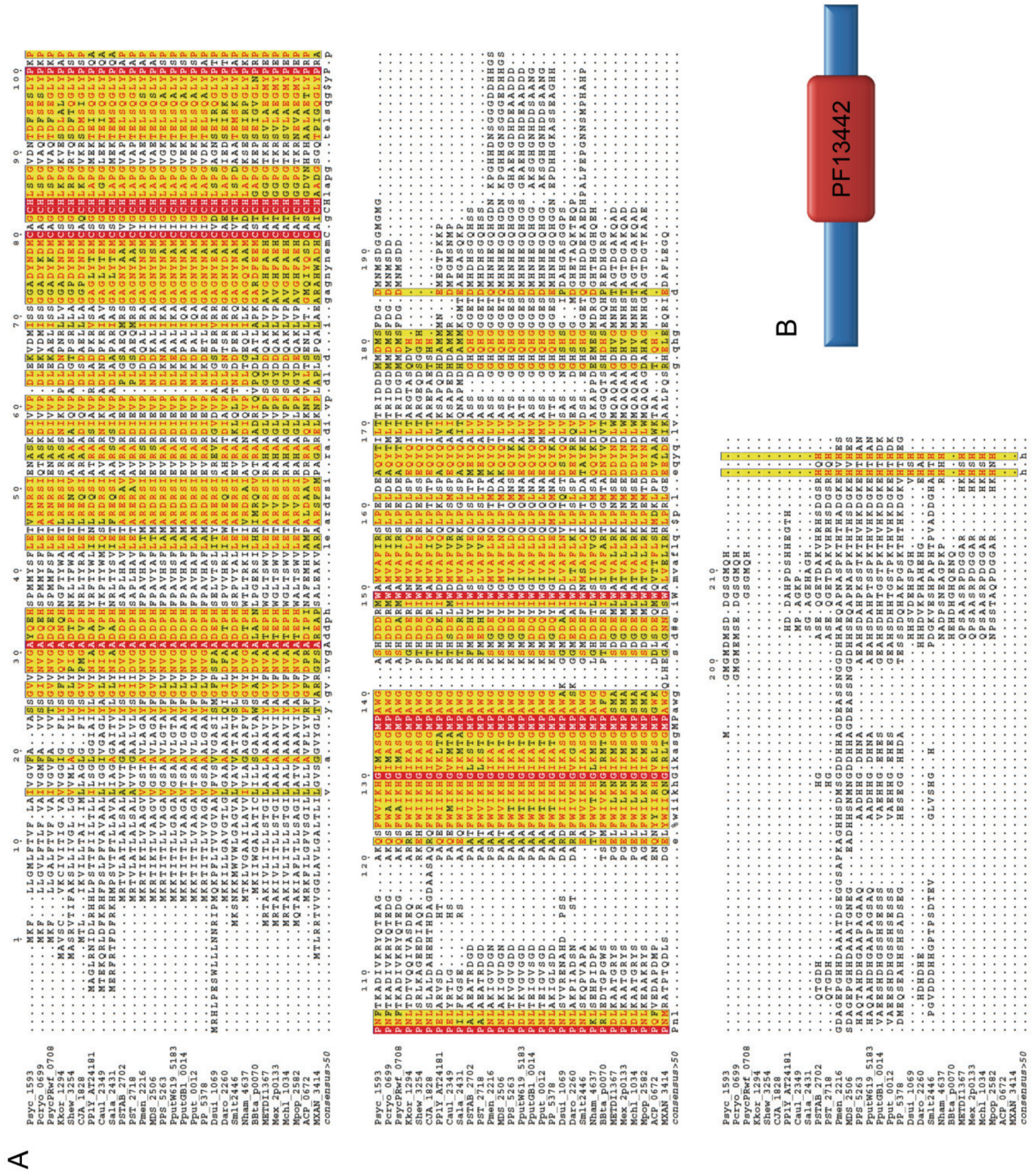

Figure S4: MXAN_3414-like proteins. A. Alignments of the 29 proteins with the conserved PF13442 (cytochrome *c* oxidase, cbb3-type, subunit III) and genetic environments with copper related proteins represented in Figure S3. **B**. Representative domain architecture
