# Supplementary material for: The Myxococcus xanthus Two-Component System CorSR Regulates Expression of a Gene Cluster Involved in Maintaining Copper Tolerance during Growth and Development
Source: PLoS One. 2013 Jul 10;8(7):e68240. doi: 10.1371/journal.pone.0068240 (PMC3707914; doi:10.1371/journal.pone.0068240)
Supplement: Table S1 — Oligonucleotides used in this study. (DOC) [file pone.0068240.s005.doc]

**Table S1.** Oligonucleotides used in this study

| Oligonucleotide | Purpose | Sequence (5’→3’)^a^ |
| --- | --- | --- |
| FRTC | Synthesis of cDNA | TGGTCCTTCCCTGTGCCCTT |
| ORTF | Amplification of DNA using cDNA as template | TGGGACTCCTTGCTGCACTC |
| ORTR | Amplification of DNA using cDNA as template | TGATGTGGTCCATCGCGCAG |
| HKLcsABamF | Amplification of upstream of *MXAN3419* (pAMΔ*corSR* and pKY481-CorSR) | CCCGGATCCAACGCTGGTGGTGTCTGGAG |
| HKLcsAPstR2 | Amplification of upstream of *MXAN3419* (pAMΔ*corSR* and pKY481-CorSR) | GACTGCAGGGGCCTCGTGGATCTCCGCC |
| RRLcsAPstF | Amplification of dowstream of *MXAN3418* (pAMΔ*corSR* ) | TCCCTGCAGTCCTCAAGCGCTTTGGCCTG |
| RRLcsAHinR | Amplification of dowstream of *MXAN3418* (pAMΔ*corSR* ) | TTGAAGCTTTCGTTGTCCACGTACATGAG |
| carBKpnI | Amplification of upstream of *carB* (pBJ113-*carB*) | CCTGGTACCCACGCTCCAGCAAACCTTGG |
| carBBamHI1 | Amplification of upstream of *carB* (pBJ113-*carB*) | GTGGGATCCATGCTTCTTTCCTCCGAAGA |
| carBBamHI2 | Amplification of downstream of *carB* (pBJ113-*carB*) | TGTGGATCCACCCGGGGGCCGAATGATAC |
| carBHindIII | Amplification of downstream of *carB* (pBJ113-*carB*) | GGCAAGCTTTCGCCGCGTGAATCACGACG |
| Mut3414KpnF | Amplification of upstream of *MXAN3414* (pBJ113-Mut3414) | TCCGGTACCAGACGCACGGCTTCAAGTC |
| Mut3414BamR | Amplification of upstream of *MXAN3414* (pBJ113-Mut3414) | CGCGGATCCACGGGGTATTCTCCTCTTG |
| Mut3414BamF | Amplification of downstream of *MXAN3414* (pBJ113-Mut3414) | CGAGGATCCGTAGCCGTCACATGTGAAAG |
| Mut3414HinR | Amplification of downstream of *MXAN3414* (pBJ113-Mut3414) | TGCAAGCTTAATGGGTGGAGGCGGTGAAT |
| MprCLacZKpnF | Amplification of upstream of MprC, *MXAN5125* (pKY481-MprC) | CGAGGTACCCTACCAACGCCTCAAGGTCAT |
| MprcLacZBamR | Amplification of upstream of MprC, *MXAN5125 (*pKY481-MprC) | AAAGGATCCATGGCATAACTCCTGGGAAGG |
| FruALacZXhoF | Amplification of upstream of FruA, *MXAN3117* (pKY481-FruA) | GACCTCGAGCACCGTCCAGTACAGCCACTG |
| FruALacZBamR | Amplification of upstream of FruA, *MXAN3117* (pKY481-FruA) | GTTGGATCCATGCGAAGGCCCCCCAGCCGT |
| tpsXhoF | Amplification of upstream of protein S*, MXAN5430* (pKY481-tps) | ccctcgagccccctgcactgggccaacc |
| tpsBamR | Amplification of upstream of protein S*, MXAN5430* (pKY481-tps) | TGGGATCCATTGCAGTGCTCCTCCGTTG |
| MXAN3421lacBam | Amplification of upstream of *MXAN3421* (pKY481-MXAN3421) | CTCGGATCCATCGCTTGGTTGCTCCCAGA |
| MXAN3421lacKpn | Amplification of upstream of *MXAN3421* (pKY481-MXAN3421) | GCCGGTACCAAGATGGGACCACCGCTGTA |

^a^ Underlined are the restriction sites used in cloning.
