# Supplementary material for: The Myxococcus xanthus Two-Component System CorSR Regulates Expression of a Gene Cluster Involved in Maintaining Copper Tolerance during Growth and Development
Source: PLoS One. 2013 Jul 10;8(7):e68240. doi: 10.1371/journal.pone.0068240 (PMC3707914; doi:10.1371/journal.pone.0068240)
Supplement: Table S2 — Bacterial strains and plasmids used in this study. (DOC) [file pone.0068240.s006.doc]

**Table S2.** Bacterial strains and plasmids used in this study.

| **Bacterial strains** | **Genotype/phenotype^a^** | **Reference or source** |
| --- | --- | --- |
| *E.coli* |  |  |
| JM109 | F´[*traD*36 *proAB*+ *lacI*q l*acZ* ΔM15] *recA*1 *supE*44 *endA*1 *hsdR*17 *gyrA*96 *relA*1 *thi* Δ(*lac*-*proAB*) | 1 |
| TOP10 | F^-^ *mcr*A Δ(*mrr*-*hsd*RMS-*mcr*BC) Φ80*lac*ZΔM15 Δ*lac*X74 *rec*A1 *ara*D139 Δ(*ara*-*leu*)7697 *gal*U *gal*K *rps*L (Str^R^) *end*A1 *nup*G | Invitrogen |
| *M. xanthus* |  |  |
| DZF1 | *pilQ1*, used as wild type | 2 |
| JM51DIF | Δ*corSR*, Gal^R^ Km^S^ | This study |
| JMcarBIF | Δ*carB*, Gal^R^ Km^S^ | This study |
| JMcarBDIF | Δ*corSR*-Δ*carB*, Gal^R^ Km^S^ | This study |
| JM300 | Δ*MXAN3414*, Gal^R^ Km^S^ | This study |
| JM301 | Δ*corSR*-Δ*corE*, Gal^R^ Km^S^ | This study |
| JM302 | Δ*corSR*-Δ*corE copA-lacZ*, Km^R^ | This study |
| JM303 | *corSR-lacZ*, Km^R^ | This study |
| JM304 | Δ*corSR*, *corSR-lacZ*, Km^R^ | This study |
| JM51AZY | *cuoA-lacZ*, Km^R^ | 3 |
| JM51DAZY | *ΔcorSR, cuoA-lacZ*, Km^R^ | This study |
| JMCAlac | *copA-lacZ*, Km^R^ | 4 |
| JM305 | *ΔcorSR, copA-lacZ*, Km^R^ | This study |
| JM306 | *mprC-lacZ* Km^R^ | This study |
| JM307 | *ΔcorSR, mprC-lacZ*, Km^R^ | This study |
| JM308 | *fruA-lacZ*, Km^R^ | This study |
| JM309 | *ΔcorSR, fruA-lacZ*, Km^R^ | This study |
| JM310 | *tps-lacZ*, Km^R^ | This study |
| JM311 | *ΔcorSR, tps-lacZ*, Km^R^ | This study |
| JM312 | *MXAN_3421-lacZ,* Km^R^ | This study |
| JM313 | *ΔcorSR, MXAN_3421-lacZ,* Km^R^ | This study |
|  |  |  |
| **Plasmids** | **Revelant features and/or genes^a^** | **Reference or source** |
| pBJ113 | *galK*, Km^R^ | 5 |
| pKY481 | *lacZY*, Km^R^ | 6 |
| pAMΔ*corSR* | Δ*corSR*, Km^R^ | This study |
| pBJ113Δ*corE* | Δ*corE*, Km^R^ | 7 |
| pBJ113-Δ*carB* | Δ*carB*, Km^R^ | This study |
| pBJ113-Δ3414 | Δ*MXAN3414*, Km^R^ | This study |
| pKY481-CorSR | *corSR-lacZ*, Km^R^ | This study |
| pKY481-CuoA | *cuoA-lacZ*, Km^R^ | 3 |
| pAELCAlac | *copA-lacZ*, Km^R^ | 4 |
| pKY481-MprC | *mprC-lacZ*, Km^R^ | This study |
| pKY481-FruA | *fruA-lacZ*, Km^R^ | This study |
| pKY481-Tps | *tps-lacZ*, Km^R^ | This study |
| pKY481-MXA_N3421 | *MXAN_3421-lacZ,* Km^R^ | This study |

^a^Km^R^ and Km^S^ mean kanamycin resistant and kanamycin sensitive, respectively. Gal^R^ indicates galactose resistance.

**REFERENCES**

1. Yanisch-Perron C, Vieira J, Messing J (1985) Improved M13 phage cloning vectors and host strains: nucleotide sequences of the M13mp18 and pUC19 vectors. Gene 33: 103-119.
2. Morrison CE, Zusman DR (1979) *Myxococcus* *xanthus* mutants with temperature-sensitive, stage-specific defects: evidence for independent pathways in development. J Bacteriol 140: 1036-1042.
3. Sánchez-Sutil MC, Gómez-Santos N, Moraleda-Muñoz A, Martins LO, Pérez J et al., (2007) Differential expression of the three multicopper oxidases from *Myxococcus xanthus*. J Bacteriol 189: 4887-4898.
4. Moraleda-Muñoz A, Pérez J, Extremera-León AL, Muñoz-Dorado J (2010b) Expression and physiological role of three *Myxococcus xanthus* copper-dependent P_1B_-type ATPases during bacterial growth and development. Appl Environ Microbiol 76: 6077-6084.
5. Julien, B, Kaiser D, Garza A (2000) Spatial control of cell differentiation in *Myxococcus xanthus*. Proc Natl Acad Sci USA 97: 9098–9103.
6. Cho K, Zusman DR (1999) AsgD, a new two-component regulator required for A-signalling and nutrient sensing during early development of *Myxococcus xanthus*. Mol Microbiol 34: 268–281.
7. Gómez-santos N, Pérez J, Sánchez-Sutil MC, Moraleda-Muñoz A, Muñoz-Dorado J (2011) CorE from *Myxococcus xanthus* is a copper-dependent RNA polymerase sigma factor. PLoS Genetics 7: e1002106.
